# Solitary Myofibroma of the Bladder Trigone in a 3-Month-Old Patient: First Case Report

**DOI:** 10.1155/2016/1951840

**Published:** 2016-05-03

**Authors:** Marco Pensabene, Fortunato Siracusa, Vito Rodolico, Giuseppe Li Voti, Elisa Zambaiti, Marcello Cimador

**Affiliations:** ^1^Pediatric Urology, Department for Health Promotion & Mother and Child Care, University of Palermo, Via Alfonso Giordano 3, 90127 Palermo, Italy; ^2^Human Pathology, Department for Health Promotion & Mother and Child Care, University of Palermo, Via Alfonso Giordano 3, 90127 Palermo, Italy

## Abstract

Visceral solitary myofibromas are uncommon in childhood. We report a case of a solitary asymptomatic visceral myofibroma of the bladder trigone occurring in a 3-month-old boy. Once malignancies were ruled out by cystoscopy, radical excision was performed in order to avoid any potential impairment of bladder dynamic. Postoperative course was uneventful and patient was discharged on day 3 after surgery. After 36 months of follow-up, the patient is toilet-trained and remains well; bladder function is normal.

## 1. Introduction

Visceral myofibromas are uncommon in childhood. They are often detected in case of infantile myofibromatosis. Solitary visceral myofibromas are exceedingly rare in children; they are congenital and usually detected within 2 years of life.

We report a case of a solitary asymptomatic visceral myofibroma of the bladder trigone occurring in a 3-month-old boy. Patient came at our institution because microhematuria was detected. During an ultrasound investigation of abdomen, a mass in the bladder trigone was detected; multiple biopsies of the mass were obtained during cystoscopic procedure and radical excision of the mass was performed 6 weeks later. Patient had a regular postoperative course and remains well after 36 months of follow-up.

To our knowledge, this is the first reported case of myofibroma occurring in this anatomic site. Despite solitary visceral myofibromas being of benign features, the anatomic site pushed us to perform a surgical excision in order to avoid any impairment of bladder function.

## 2. Case Presentation

**Figure 1:**
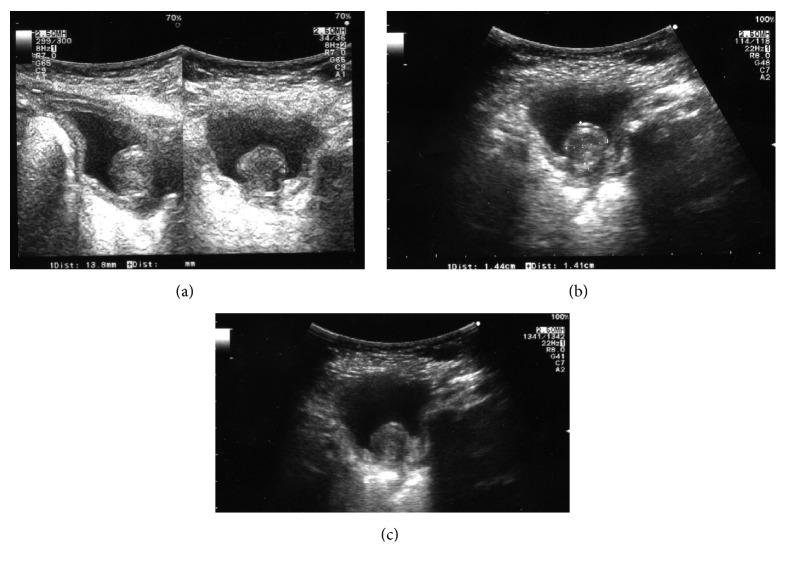
Ultrasound appearance of the mass: the solitary myofibroma arises from the bladder neck gaining the bladder lumen; (a–c): longitudinal and transverse US sections.

**Figure 2:**
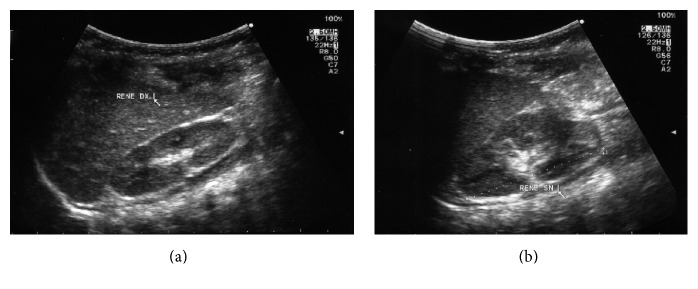
Renal ultrasound: absence of hydronephrosis was showed during a complete abdomen US examination; (a) right kidney and (b) left kidney.

A 3-month-old male patient came at our institution with a recent history of microhematuria detected by urine analysis performed for other reasons. The patient appeared in good general condition. No other clinical signs or symptoms were present, and neither was urinary tract infection. An ultrasound (US) investigation of abdomen was performed and a bladder solitary mass was detected (Figure 1). The mass was regular and grossly hypoechogenic, measuring 1,2 × 1,5 cm at US; it appeared as an exophytic mass protruding into the visceral lumen, originating from the bladder trigone, overhanging the bladder neck. US investigation excluded hydronephrosis (Figure 2) or other lesions in the abdomen, neck, and head.

We decided to perform operative cystoscopy in order to obtain multiple biopsies, allowing us to rule out malignancies. Bladder wall was normal on cystoscopic exploration, and biopsies of the mass were easily obtained. Biopsies showed a low mitotic index and uniform cells, with an eosinophil cytoplasm; no evidence of malignancy was present.

**Figure 3:**
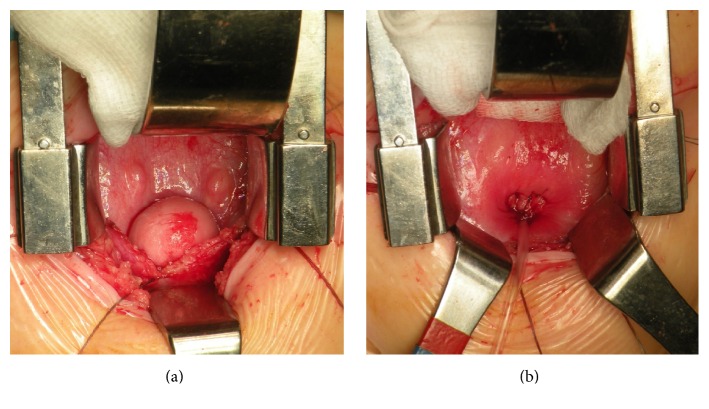
Intraoperative images. (a) Intraoperative appearance of the vesical trigone, ureteral orifices, and the mass occupying the bladder neck; (b) the mass is removed and the bladder mucosa sutured. An indwelling catheter is inserted as urethral tutor.

Thus, surgery excision of the mass was performed 6 weeks later via a mini-Pfannenstiel. The mass was completely excised (Figure 3). Postoperative course was uneventful, a Foley catheter was left in the bladder during first 24 hours, and the patient was discharged on postoperative day 3.

He remains well after 36 months of follow-up; actually, the patient showed normal urinalysis: no microhematuria, pH 5.5, specific gravity 1010, and no crystals or casts; UTI were excluded because no nitrites and leukocyte esterase were found, and less than 15 WBCs/hpf were observed.

**Figure 4:**
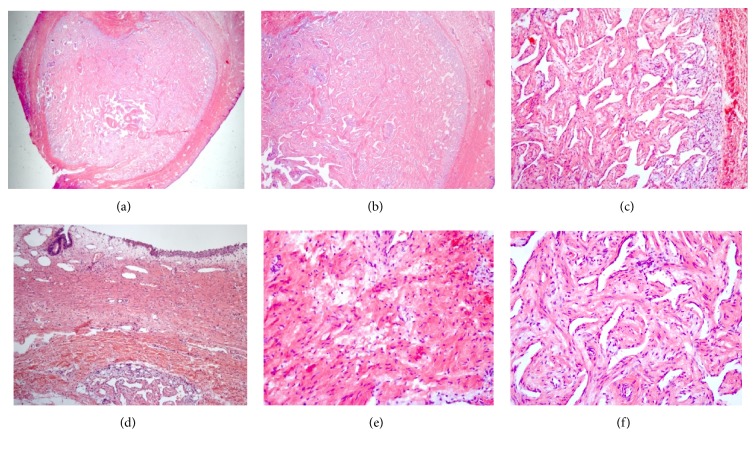
Pathology. (a–d) (*Haematoxylin/eosin*): nodular proliferation with superficial normal urothelium; (e) peripheral zones have myoid short fascicles/whorls/nodules of plump myofibroblasts with pale pink cytoplasm and long, tapering nuclei with vesicular chromatin and 1-2 small nucleoli, but no atypia or pleomorphism, often associated with hyalinization, and whorls/nodules can have a vaguely chondroid or chondromyxoid appearance; (f) central zones between the peripheral myoid nodules display cellular areas of round, polygonal, or spindle cells with scant cytoplasm, hyperchromatic nuclei, arranged around thin walled branching ectatic “hemangiopericytic” vessels; often calcification, necrosis, and hyalinization; often apparent subendothelial intravascular growth but still benign with minimal mitotic activity.

*Pathology.* The nodular well-circumcised mass measured 2 cm in diameter and was covered by normal urothelial tissue (Figure 4). Cross section of the mass showed a white fibrous tissue, without necrotic or hemorrhage areas. Microscopic evaluation revealed bland uniform cells, with an eosinophil cytoplasm, without atypical manifestations. Mitotic index was low.

Immunohistochemical stains were performed, revealing positivity for CD34. Neurofibroma and perineurioma were excluded because of negativity for S100. Histopathological findings, therefore, were oriented to “benign infantile myofibroma.”

## 3. Discussion

Solitary myofibromas are rare in children.

These lesions occur as a solitary mass in 75% of cases; if multiple lesions are detected, the feature is identified as* infantile myofibromatosis.* Most often skin and subcutaneous tissue of head and neck are involved.

Visceral lesions are exceedingly rare, while only few cases described occur in the urogenital system [1–4]. To our knowledge, this is the first reported case of myofibroma occurring in the bladder.

Solitary infantile myofibromas are congenital, and 90% of cases are detected within 2 years of life, although adult occurrence has been reported [4]. Visceral lesions are detected in 35% of the cases if multiple cutaneous lesions are present and this condition correlates with a severe prognosis due to gastrointestinal or cardiopulmonary complications [5]. Isolated intestinal myofibroma was occasionally reported as a rare cause of neonatal intestinal obstruction by Saguem et al. [6]. Anyhow, if visceral involvement is excluded, the condition is usually self-limited and spontaneously tends to regress; in these cases, the prognosis is excellent. When a single visceral lesion is detected, the prognosis is commonly excellent, with a low tendency to recurrence after surgical excision [7].

Despite the good behavior of the feature, complete excision of visceral myofibromas should be performed in children, in order to detect malignancies and to avoid any mass-related signs or symptoms.

The differential diagnosis should include sarcomas, neuroblastomas, primary or metastatic, neurofibromas, and inflammatory myofibroblastic tumor. The last is an uncommon entity with variable clinical behavior, rarely showing malignant transformation, as recently reported by Wang et al. [8].

Thus, histopathological investigation is mandatory.

In our case, the mass was easily approachable cystoscopically, and biopsies were promptly performed, allowing us to exclude malignancies.

Nevertheless, surgical excision was performed in our patient not only to exclude for certain any malignancies but also to avoid any distortion of the normal bladder trigone. The exophytic mass, in fact, protruded into the visceral lumen from the bladder trigone, overhanging the bladder neck (Figures 1 and 2), allowing a possible impairment of bladder dynamics.

The radical excision was easily obtained, and a Foley catheter was left in situ for subsequent 24 h, and the patient was discharged on postoperative day 3.

US investigation was performed once per month in the first year and then every 6 months. Neither urinary tract infection nor hematuria was detected during follow-up.

Our patient is toilet-trained now, and a normal bladder function is present after 36 months of follow-up.

In conclusion, even if uncommon, myofibroma should be considered in differential diagnosis of pediatric bladder masses. Once malignancies are excluded, surgical excision should be performed in case of any potential mass-related impairment of bladder dynamic.

## Abbreviations

US: Ultrasound
UTI: Urinary tract infection.
